# New results of partially total fuzzy graph

**DOI:** 10.1186/s13104-023-06397-w

**Published:** 2023-08-11

**Authors:** Fekadu Tesgera Agama, V. N. SrinivasaRao Repalle, Laxmi Rathour

**Affiliations:** 1 Department of Mathematics, Wollega University, Nekemte, Ethiopia; 2 Ward Number-16, Bhagatbandh, Anuppur, Madhya Pradesh 484224 India

**Keywords:** Fuzzy relation, Fuzzy sets, Partially total fuzzy graph, Fuzzy graphs, Primary 05C72, Secondary 03B20

## Abstract

**Objective:**

The study of total fuzzy graphs in all cases is crucial for the development of both theories and applications of the graph theory. Without theory the application will not be developed. Hence this manuscript attempted to theorize the conception of partially total fuzzy graphs.

**Results:**

The article introduced the partially total fuzzy graph by keeping all the conditions of fuzziness as it is. From these definitions, it is endeavored to get the partial total fuzzy graph of a given fuzzy graph which is supported by illustrations. Also, some propositions and theorems related to this concept were developed and proved.

## Introduction

One of immensely expedient in modeling is graph theory. Modeling with graphs is important in telecommunication networking problems of communication, trafficking networks and much more. Theoretical modeling with graphs provides an important structure for which analytical methods are used. For a given set of objects, graph can be used to exemplary that the relationship exists amongst them.

The announcement about uncertainty of real life is due to Zadeh [1] in his pamphlet of inspiring paper. A fuzzy relation and set is demarcated scientifically by conveying to all likely detached in the cosmos of treatise are a worth, demonstrating its grade of affiliation, which matches to the point, to which that specific is similar or well-matched with the notion characterized by the fuzzy set. Fuzzy graph is make known to us by Rosenfeld [2] using fuzzy relation, exemplifies the bond amongst the objects by surely representing the near of the relationship among the items of the given set. Also he devised many fuzzy equivalent graph hypothetical concepts like connection, cut vertex and tree.

Bhattacharya [3] and Bhutani [4] discoursed about automorphism of fuzzy graphs and announced the idea of strangeness and center in fuzzy graphs. Mordeson [5] familiarized fuzzy line graph. Mordeson and Peng [6] dispensed with the operations of fuzzy graphs and their possessions. Mordeson and Nair [7] considered cycles and co-cycles and gave a definition of counterpart of a fuzzy graph. Sunitha and Vijayakumar [8] defined the complement of a fuzzy graph in another way which gives a better sympathetic about that thought. A study on total fuzzy graph was introduced by Nagoor Gani and Rahman [9]. 2-quasi TFG, their definition, properties, and its coloring are well studied by Fekadu Tesgera Agama and V.N. Srinivasa Rao Repalle [10].

In this manuscript, we need to state the notion of partially total fuzzy graph. The graph is partially total, but not fully total fuzzy graph. With this definition we illustrate the perception with graphical example and new outcomes arising from this definition are quantified and evidenced in a logical manner.

## Preliminaries

Under this sub-topic we present definitions of fuzzy graphs and $TFG$ which are necessary for this article.

### Definition 1

[11]: A fuzzy graph $\mathrm{G}:(\mathrm{V},\upsigma ,\upmu )$ is a triple consisting of a non-empty set $\mathrm{V}$ along with the roles $\upsigma :\mathrm{V }\to [0, 1]$ and $\upmu :\mathrm{ V x V }\to [0, 1]$ such that$\forall \mathrm{u},\mathrm{ v}\in \mathrm{ V}$, $\upmu \left(\mathrm{u},\mathrm{ v}\right)\le\upsigma \left(\mathrm{u}\right)\wedge\upsigma \left(\mathrm{v}\right),$ where ∧ denotes the smallest.

### Definition 2

[11]: Let $G:(\sigma , \mu )$ be a fuzzy graph. The $order$ of $G$ is defined as: $Order\left(G\right)=\sum_{u\in V}\sigma \left(u\right)$ and the $Size$ of $G$ defined as, $Size\left(u\right)=\sum_{u,v\in V}\mu (u,v)$.

### Definition 3

[12]: A fuzzy graph $G$ is called a strong fuzzy graph if $\mu (a, b) = \sigma (a)\Lambda \sigma (b)$ for all $(a, b)$ in edge set.

### Definition 4 

[11]: A fuzzy graph $G$ is said to be a complete fuzzy graph if $\mu (a, b)$ is $\sigma (a)\Lambda \sigma (b)$ $\forall a, b$ in $\sigma ;$ where $\sigma$ is called the fuzzy vertex set of G and $\mu$ the fuzzy edge set of G.

### Definition 5

 [11]: The busy value of a $a\in V$ in $G$ is $D\left(a\right)= \sum_{i}\sigma (a)\mathrm{\Lambda \sigma }({a}_{i})$ where $a_{i}{^\prime} s$ are the neighbours of $a$ and the busy value of $G$ is $D(G)=\sum_{i}D({a}_{i})$ where $a_{i}{^\prime} s$ are in $V$ of $G$.

### Definition 6

 [5]: Let $G: (\sigma , \mu )$ be a fuzzy graph with the primary graph$(V, E)$. The fuzzy line graph of $G$ is $L(G):(\gamma , \omega )$ with the underlying graph $(Z, W)$ where$Z=\{Sa =\{a\}\cup \boldsymbol{ }\{{u}_{a}, {v}_{a}\}/x\in E, {u}_{a}, {v}_{a}\in V$, $a= ({u}_{a}, {v}_{a})\}$ with $\gamma \left(Sa\right)= \mu \left(a\right),\forall Sa\in Z$ and $W= \{(Sa,Sb) / Sa \cap Sb \ne \varnothing , a, b\in E, a\ne \boldsymbol{ }b\}$ with $w(Sa, Sb)=\omega (Sa)\Lambda \omega (Sb)= \mu (a)\Lambda \mu (b), \forall (Sa, Sb ) \in W.$.

### Definition 7

 [11]: **S**uppose $G:\left(\sigma ,\mu \right)$ is a fuzzy graph. The Total fuzzy graph is a pair $T\left(G\right)=({\sigma }_{T},{\mu }_{T})$ of $G$ where;

i. ${\sigma }_{T}$ be expressed over $V\cup E$ such that ${\sigma }_{T}\left(u\right)=\sigma \left(u\right), if\,u\in V$, ${\mu }_{T}\left(e\right)=\mu \left(e\right), if,e\in E$.
ii. ${\mu }_{T}$ is described as;
  $${\mu }_{T}\left(u,v\right)=\mu \left(u,v\right), if\,u,v\in V,$$

$${\mu }_{T}\left(u,e\right)=\sigma \left(u\right)\Lambda \mu \left(e\right), if u\in V, e\in E,$$ and the vertex $$u$$ lies on the edge $$e,$$$${\mu }_{T}\left(u,e\right)=0$$, otherwise,$${\mu }_{T}\left({ e}_{i},{ e}_{j}\right)=\mu \left({e}_{i}\right)\Lambda \mu \left({e}_{j}\right),$$ if the edges $${e}_{i}$$ and $${e}_{j}$$ have a node in common between them, $${\mu }_{T}\left( {e}_{i}, {e}_{j}\right)=0,$$ otherwise.

## Main text

### The properties of partially total fuzzy graph

#### Definition 8

Suppose $G:(\sigma ,\mu )$ is a fuzzy graph. Let $V$ be the primary set. Assume that ${G}^{*}:({\sigma }^{*},{\mu }^{*})$ is its crisp graph. Considering the node set of ${P}_{T}(G)$ be the combination of vertex set and edge set of $G$, we will define ${P}_{T}(G)$ on $V\cup E$ according to the following. ${\sigma }_{P}\left(a\right)=\sigma \left(a\right),$ whenever $a\in V$ $=\mu \left(x\right),$ whenever $x\in E$. For ${\mu }_{P}$, we define it as below;

$${\mu }_{P}\left(a,b\right)=0, \mathrm{whenever }\,a,b\in V$$

$${\mu }_{P}\left(a,x\right)=\sigma (a)\bigwedge \mu (x)$$, whenever $$a\in V$$,$$x\in E$$ and $$a$$ lies on $$x$$$$=0$$, otherwise. $${\mu }_{P}\left({x}_{i},{x}_{j}\right)=\mu ({x}_{i})\bigwedge \mu ({x}_{j})$$, whenever $${x}_{i},{x}_{j}\in E$$, and they have a vertex in common. $$=0$$, otherwise.

According to this definition, we can observe that ${\mu }_{P}\left(a,b\right)\le \sigma (a)\bigwedge \sigma (b)$, $\forall a,b\in V\cup E$. This implies that the pair ${P}_{T}\left(G\right):({\sigma }_{P},{\mu }_{P})$ represents a fuzzy graph and we name such graph partially total fuzzy graph of a fuzzy graph $G$.

#### Example 1

Given graph $G$ with the following information.$\sigma \left(a\right)=0.4$, $\sigma \left(b\right)=0.5$, $\sigma \left(c\right)=0.7$ and $\mu \left(a,b\right)=0.2$,$\mu \left(b,c\right)=0.4$,$\mu \left(a,c\right)=0.4$.

From the given information, one can deduce that the vertex set is $\{a,b,c\}$ and the edge set is $\{ab,bc,ac\}$. Thus, it can be verified that $\mu (a,b)\le \sigma (a)\bigwedge \sigma (b)$, $\forall a,b\in V$. Therefore, the given graph is a fuzzy graph. Moreover, as there is an edge between every pair of elements of the node set, and then it is complete fuzzy graph. Our objective is to define a partially total fuzzy graph ${P}_{T}\left(G\right)$ for this fuzzy graph. To do so, we start from finding the node set which is $V\cup E=\{a,b,c,ab,bc,ac\}$. From the definition of ${P}_{T}\left(G\right)$ the fuzzy set is defined as; ${\sigma }_{P}\left(a\right)=\sigma \left(a\right),$ whenever $a\in V$

$$=\mu \left(x\right),$$ whenever $$x\in E$$.

Hence; we have the following fuzzy subset.

$$\sigma \left(a\right)=0.4, \sigma \left(b\right)=0.5,$$

$$\sigma \left(c\right)=0.7,$$

$$\sigma \left(ab\right)=\mu \left(a,b\right)=0.2,\,\sigma \left(bc\right)=\mu \left(b,c\right)=0.4,\,\sigma \left(ac\right)=\mu (a,c)=0.4,$$

The fuzzy relation for this ${P}_{T}\left(G\right)$ becomes;

$$\mu \left(a,ab\right)=\sigma \left(a\right)\bigwedge \mu \left(a,b\right)=0.2, \mu \left(a,ac\right)=\sigma \left(a\right)\bigwedge \mu \left(a,c\right)=0.4$$

$$\mu \left(b,ab\right)=\sigma \left(b\right)\bigwedge \mu \left(a,b\right)=0.2, \mu \left(b,bc\right)=\sigma \left(b\right)\bigwedge \mu \left(b,c\right)=0.4$$

$$\mu \left(c,ac\right)=\sigma \left(c\right)\bigwedge \mu \left(a,c\right)=0.4, \mu \left(c,bc\right)=\sigma \left(c\right)\bigwedge \mu \left(b,c\right)=0.4$$

$$\mu \left(ab,bc\right)=\mu \left(a,b\right)\bigwedge \mu \left(b,c\right)=0.2, \mu \left(ab,ac\right)=\mu \left(a,b\right)\bigwedge \mu \left(a,c\right)=0.2$$

$$\mu \left(bc,ac\right)=\mu \left(b,c\right)\bigwedge \mu \left(a,c\right)=0.4,$$

Clearly, $\mu \left(a,b\right)=\sigma \left(b\right)\bigwedge \sigma \left(b\right)$, $\forall a,b\in V\cup E$. This justifies that ${P}_{T}\left(G\right)$ is a fuzzy graph and termed as partially total fuzzy graph of the given fuzzy graph $G$.

**Fig. 1:**
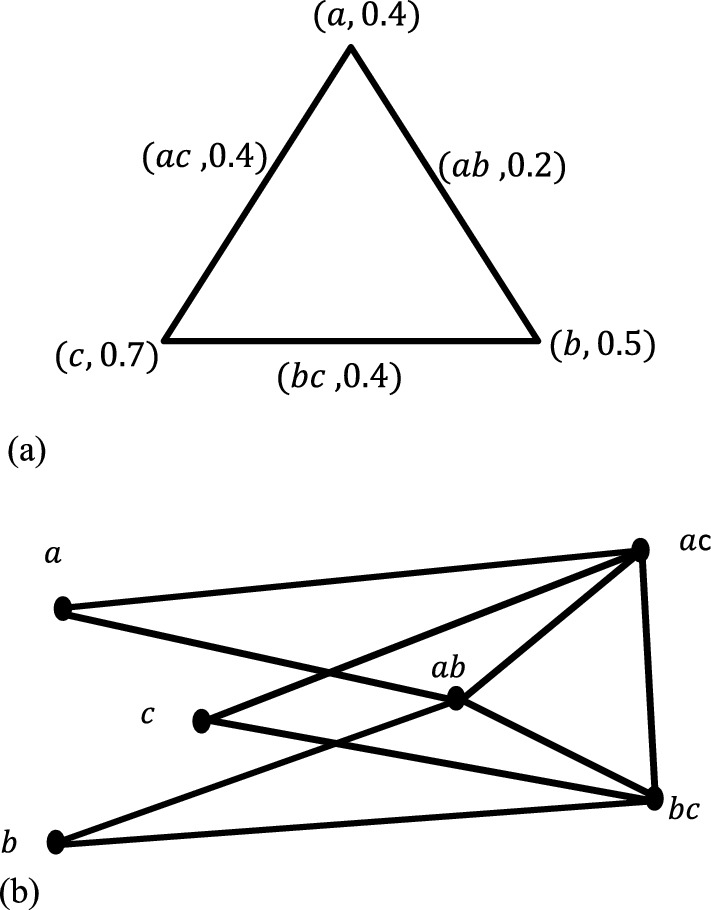
**a**: $$G:(\sigma ,\mu )$$. **b**: $${P}_{T}:({\sigma }_{P},{\mu }_{G})$$

The following figures Fig. 1a, b represents a fuzzy graph $G$ and its partially total fuzzy graph.

#### Example 2

**Fig. 2:**
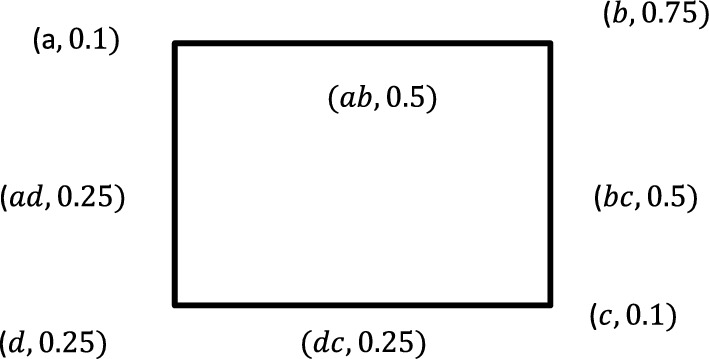
Fuzzy Graph $$G$$

Given the following fuzzy graph as shown in Fig. 2.

The objective is to define and sketch the partially total fuzzy graph for this fuzzy graph $G$. To do this we need to determine the vertex set of ${P}_{T}(G)$. It is $\{a,b,c,d,ab,bc,cd,da\}$ with fuzzy subset ${\sigma }_{P}$ becomes;

$$\sigma \left(a\right)=0.1, \sigma \left(b\right)=0.75, \sigma \left(c\right)=0.1, \sigma \left(d\right)=0.25,$$

$$\sigma \left(ab\right)=\mu (a,b)=0.5,$$

$$\sigma \left(bc\right)=\mu (b,c)=0.5,$$

$$\sigma \left(cd\right)=\mu \left(c,d\right)=0.25,\,\sigma \left(da\right)=\mu (d,a)=0.25$$

The fuzzy relation for ${P}_{T}(G)$ will be;

$$\mu \left(a,ab\right)=\sigma \left(a\right)\bigwedge \mu \left(a,b\right)=0.25,\,\mu \left(a,ad\right)=\sigma \left(a\right)\bigwedge \mu \left(a,d\right)=0.2s,$$

$$\mu \left(b,ab\right)=\sigma \left(b\right)\bigwedge \mu \left(a,b\right)=0.2s,\,\mu \left(b,bc\right)=\sigma \left(b\right)\bigwedge \mu \left(b,c\right)=0.5,$$

$$\mu \left(c,bc\right)=\sigma \left(c\right)\bigwedge \mu \left(b,c\right)=0.5,\,\mu \left(c,cd\right)=\sigma \left(c\right)\bigwedge \mu \left(c,d\right)=0.25,$$

$$\mu \left(d,cd\right)=\sigma \left(d\right)\bigwedge \mu \left(c,d\right)=0.25,\,\mu \left(d,ad\right)=\sigma \left(dd\right)\bigwedge \mu \left(a,d\right)=0.25$$

$$\mu \left(ab,bc\right)=\mu \left(a,b\right)\bigwedge \mu \left(b,c\right)=0.5,\,\mu \left(bc,cd\right)=\mu \left(b,c\right)\bigwedge \mu \left(c,d\right)=0.25,$$

$$\mu \left(cd,da\right)=\mu \left(c,d\right)\bigwedge \mu \left(d,a\right)=0.25, \mu \left(da,ab\right)=\mu \left(d,a\right)\bigwedge \mu \left(a,b\right)=0.25$$

**Fig. 3:**
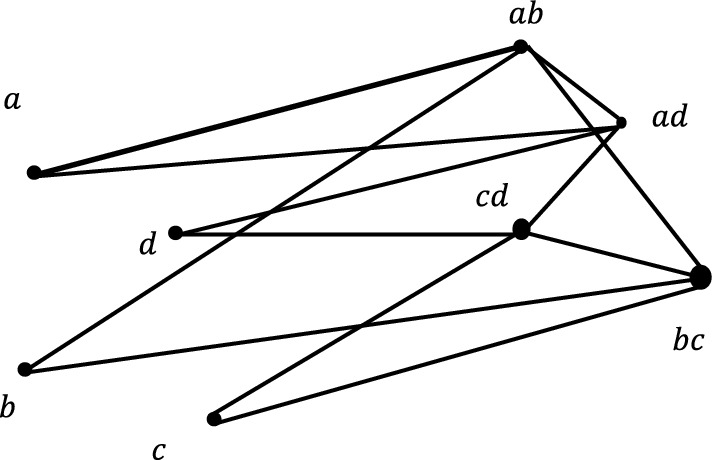
Partial Total Graph of Fuzzy Graph $G$

It is observable that,$\mu \left(a,b\right)=\sigma \left(b\right)\bigwedge \sigma \left(b\right)$, $\forall a,b\in V\cup E$. Therefore, ${P}_{T}\left(G\right)$ is a fuzzy graph its graph is as given in Fig. 3.

## Results from partially total fuzzy graph

### Theorem 1

The order of partially total fuzzy graph of $G$ is the sum of the order of $G$ and the size of $G$.

### Proof

We know that the node set of ${P}_{T}\left(G\right)$ is $V\cup E$ and ${\sigma }_{P}$ which is defined on $V\cup E$ is; ${\sigma }_{P}\left(a\right)=\sigma \left(a\right),$ whenever $a\in V$ $=\mu \left(x\right),$ whenever $x\in E$.

We can recall that $\mathrm{order}\left({P}_{T}\left(G\right)\right)=\sum_{a\epsilon V\cup E}{\sigma }_{P}(a)$, which is obtained by definition from order of fuzzy graph.

Hence, $$order\left({P}_{T}\left(G\right)\right)=\sum_{a\epsilon V\cup E}{\sigma }_{P}(a)$$

$$=\sum_{a\epsilon V}{\sigma }_{P}(a)+\sum_{a\epsilon E}{\sigma }_{P}(a)$$

$$=\sum_{a\epsilon V}\sigma (a)+\sum_{a\epsilon E}\mu (a)$$

$$=order\left(G\right)+Size(G) $$

$$\square $$

### Theorem 2

The size of a partial TFG is two times the size of $G$ plus $\sum_{{x}_{i}, {x}_{j}\epsilon E}\mu ({x}_{i})\bigwedge \mu ({x}_{j})$.

### Proof

$$Size\left({P}_{T}\left(G\right)\right)=\sum_{a,b\epsilon V\cup E}{\mu }_{P}(a,b)$$

$$=\sum_{a,b\epsilon V}{\mu }_{P}(a,b)+\sum_{a\epsilon V,x\in E}{\mu }_{P}(a,x)+\sum_{{x}_{i},{x}_{j}\epsilon V\cup E}{\mu }_{P}({x}_{i},{x}_{j})$$

In this new definition of the concept of partially TFG, there is no fuzzy relation between any two vertices of the graph $G$. Hence, $\sum_{a,b\epsilon V}{\mu }_{P}(a,b)=0.$

Thus, $$Size\left({P}_{T}\left(G\right)\right)=\sum_{a\epsilon V,x\in E}{\mu }_{P}(a,x)+\sum_{{x}_{i},{x}_{j}\epsilon V\cup E}{\mu }_{P}({x}_{i},{x}_{j})$$

$$=\sum_{a\epsilon V,x\in E}\mu (a,x)+\sum_{{x}_{i},{x}_{j}\epsilon V\cup E}\mu ({x}_{i},{x}_{j})$$

It is a clear fact that two vertices lie on each edge and its weight is less than the weight of the vertices, we have $\sum_{a\epsilon V,x\in E}\mu (a,x)=2\sum_{x\in E}\mu (x)$.

Therefore, $$Size\left({P}_{T}\left(G\right)\right)=2\sum_{x\in E}\mu (x)+\sum_{{x}_{i},{x}_{j}\epsilon V\cup E}\mu \left({x}_{i}\right)\bigwedge \mu ({x}_{j})$$

$$=2Size\left(G\right)+\sum_{{x}_{i},{x}_{j}\epsilon V\cup E}\mu \left({x}_{i}\right)\bigwedge \mu ({x}_{j})$$

$$\square $$

### Theorem 3

The degree of $a$ of ${P}_{T}(G)$ is the same as the degree of $a$ in $G$, if $a\in V$ and that of $x\in E$ is equal to the busy value of $x$ in ${P}_{T}(G)$.

### Proof

The nodes of ${P}_{T}(G)$ are from the vertices and edges of $G$. Accordingly, the determination of their degree needs separate treatment.

Case $i$: Let $a\in V$. ${d}_{P\left(G\right)}\left(a\right)=\sum_{b\in V}{\mu }_{P}\left(a,b\right)+\sum_{x\in E}{\mu }_{P}\left(a,x\right)$, where $a$ lies on the edge$x\in E$.

$=\sum_{x\in E}{\mu }_{P}\left(a,x\right)$, as there is no fuzzy relation between any two vertices of $G$ in ${P}_{T}(G)$.

$$=\sum_{x\in E}\mu \left(x\right)={d}_{G}(a) $$

$$\square $$

Case $$ii$$: Let $${x}_{i}\in E$$.

$${d}_{P\left(G\right)}\left({x}_{i}\right)=\sum_{a\in V}{\mu }_{P}\left({x}_{i},a\right)+\sum_{{x}_{j}\in E}{\mu }_{P}\left({x}_{i},{x}_{j}\right)$$

$$=\sum_{a\in V}\mu \left({x}_{i})\bigwedge \sigma (a\right)+\sum_{{x}_{j}\in E}\mu \left({x}_{i}){\bigwedge \sigma (x}_{j}\right)$$

$=$ busy value of ${x}_{i}$ in ${P}_{T}(G)$

$$\square $$

### Theorem 4

The number of edges in ${P}_{T}(G)$ is twice the edges in $G$ plus the number of pair wise adjacent edges in $G$.

### Proof

As each edge in $G$ is contributing two edges to ${P}_{T}(G)$ and the pair of adjacent edges in $G$, contribute an edge to ${P}_{T}(G)$, we have;

Number of edges in ${P}_{T}\left(G\right)=2\left| E\left(G\right) \right|+$ No. of pair wise adjacent edges in $G$.

$$=2\left|E(G)\right|+\left|E(L(G)\right| $$

$$\square $$

### Theorem 5

${P}_{T}\left(G\right)$ is strong fuzzy graph.

### Proof

Consider and edge $(a,b)\in {P}_{T}\left(G\right)$.

Then, ${\mu }_{P}\left(a,b\right)=\mu \left(a)\bigwedge \mu (b\right)$, if $a$ in ${\sigma }^{*}$ lies on the edge $b={x}_{j}\in {\mu }^{*}$ and.

$=\mu \left({x}_{i})\bigwedge \mu ({x}_{j}\right)$, if $a={x}_{i}$, $b={x}_{j}\in {\mu }^{*}$ and are adjacent in ${G}^{*}$.

Relating this expression to the definition of partially total fuzzy graph, we get;${\mu }_{P}\left(a,b\right)=\mu \left(a)\bigwedge \mu (b\right)$, that is a strong fuzzy graph

$$\square $$

### Theorem 6

Partially total fuzzy graph of a complete fuzzy graph is not complete fuzzy graph.

### Proof

Suppose $G$ is a complete fuzzy graph. Then every pair of vertices are adjacent in ${G}^{*}$. But from the definition of ${P}_{T}\left(G\right)$, no two nodes in $V(G)$ are adjacent to each other in ${P}_{T}\left(G\right)$. This shows that there is no edge between any vertices of ${P}_{T}\left(G\right)$ which are vertices for $G$. Hence ${P}_{T}\left(G\right)$ is not complete fuzzy graph.

### Theorem 7

Busy value of ${P}_{T}\left(G\right)=4\left(Size\left(G\right)\right)+2(\sum_{{x}_{i},{x}_{j}\in {\mu }^{*}}\mu \left({x}_{i}\right)\bigwedge \mu \left({x}_{j}\right))$.

### Proof

Busy value of ${P}_{T}\left(G\right)=\sum_{a\in V\cup E}D(a)$

$=\sum_{a\in V\cup E}d(a)$, since ${P}_{T}\left(G\right)$ is strong fuzzy graph, then $D\left(a\right)=d(a).$

$$=2(Size{P}_{T}\left(G\right))$$

$$=2(2(Size\left(G\right))+\sum_{{x}_{i},{x}_{j}\in {\mu }^{*}}\mu \left({x}_{i}\right)\bigwedge \mu \left({x}_{j}\right))$$

$$=4\left(Size\left(G\right)\right)+2(\sum_{{x}_{i},{x}_{j}\in {\mu }^{*}}\mu \left({x}_{i}\right)\bigwedge \mu \left({x}_{j}\right)) $$

$$\square $$

## Limitations

In this article, the researchers are motivated to establish a new theory of fuzzy graphs with the intension to investigate the application area for the idea. Here we have presented the new theory of partially total fuzzy graph. For this new theory we have given the definition and based on this definition partially total fuzzy graphs are discussed with examples. In addition to this we have presented some properties which arise from the definition of the concept under study. These properties are stated and systematically we have proved the theorems related with the emerging of this new concept.

## Data Availability

The authors declare that the data supporting the findings are included in the paper.
